# Estimating Zika Virus Seroprevalence in a Dengue Virus–Endemic Population: The Use of Blood Donors and Multiplex Serology to Monitor Arbovirus Outbreaks in the Dutch Caribbean

**DOI:** 10.1093/infdis/jiaf281

**Published:** 2025-05-27

**Authors:** Louella M R Kasbergen, Reina S Sikkema, Felicity Chandler, Janko H G van Beek, Yaskara Halabi, Norédiz Lourents, Eugene G Maduro, Izzy Gerstenbluth, Ashley Duits, Marion P G Koopmans

**Affiliations:** Department of Viroscience, Erasmus University Medical Center, Rotterdam, The Netherlands; Department of Viroscience, Erasmus University Medical Center, Rotterdam, The Netherlands; Department of Viroscience, Erasmus University Medical Center, Rotterdam, The Netherlands; Department of Viroscience, Erasmus University Medical Center, Rotterdam, The Netherlands; Epidemiology and Research, Ministry of Health, Environment and Nature, Willemstad, Curaçao; Epidemiology and Research, Ministry of Health, Environment and Nature, Willemstad, Curaçao; Curaçao Public Health Service (GGD), Ministry of Health, Willemstad, Curaçao; Epidemiology and Research Unit, Department of Public Health Aruba, Oranjestad, Aruba; Epidemiology and Research, Ministry of Health, Environment and Nature, Willemstad, Curaçao; Curaçao Public Health Service (GGD), Ministry of Health, Willemstad, Curaçao; Department of Epidemiology and Immunology, Curaçao Biomedical and Health Research Institute, Willemstad, Curaçao; Department of Immunohematology, Red Cross Blood Bank Foundation Curaçao, Willemstad, Curaçao; Institute for Medical Education, University Medical Center Groningen, Groningen, The Netherlands; Department of Epidemiology and Immunology, Curaçao Biomedical and Health Research Institute, Willemstad, Curaçao; Department of Immunohematology, Horacio Oduber Hospital Blood Bank, Oranjestad, Aruba; Department of Viroscience, Erasmus University Medical Center, Rotterdam, The Netherlands

**Keywords:** arbovirus, Zika virus, dengue virus, seroprevalence, Caribbean

## Abstract

**Background:**

The geographic range of flaviviruses is expanding, as evidenced by the increase in dengue virus (DENV) cases and the recent emergence of Zika virus (ZIKV) in the Americas. Studying seroprevalence of flaviviruses in (hyper)endemic regions is challenging due to extensive antibody cross-reactivity. Over the past decade, Aruba and Curaçao have experienced DENV and ZIKV outbreaks, of which the magnitude and impact remain unclear due to the challenges of disentangling DENV from ZIKV (past) infection.

**Methods:**

Here, we study the potential utility of blood donors for monitoring the extent of circulation, on 2 Caribbean islands. We report a longitudinal cross-sectional study using randomly selected blood donor sera (n = 715) during the ZIKV outbreaks on Aruba and Curaçao (January 2016–July 2017). Flavivirus seroprevalence was estimated using a validated multiplex protein microarray containing NS1 proteins for 11 flaviviruses, in combination with confirmation virus neutralization tests on a selection of sera.

**Results:**

It was possible to disentangle ZIKV exposure from exposure to other flaviviruses. In Curaçao, the seroprevalence of ZIKV antibodies was 25% and significantly higher than the prevalence of antibodies attributable to ZIKV exposure in Aruba (8%). Flavivirus immunoglobulin G antibody (IgG) titers increased with age (*P* < .05),

**Conclusions:**

This methodology allows estimation of ZIKV seroprevalence during and after the ZIKV outbreaks. This study shows the importance of including age in the interpretation of serology data. Furthermore, it gives insights into the complex antibody (background) patterns of flaviviruses over time, and highlights the utility of an existing voluntary blood donor program to monitor and respond to (flavivirus) outbreaks.

## Teaser

Using blood donor sera from Aruba and Curaçao during the Zika virus (ZIKV) outbreaks, in combination with multiplex serology and confirmation virus neutralization assays, it is possible to distinguish ZIKV exposure from other flavivirus exposures and estimate ZIKV seroprevalence.

Arthropod-borne arboviruses increasingly (re)emerge globally due to, for example, increased travel and trade and climate change [1]. A recent example is the emergence of Zika virus (ZIKV), a flavivirus that is associated with severe disease such as Guillain-Barré syndrome and microcephaly in children infected in utero [2]. Over a period of approximately 7 years, ZIKV spread to the Pacific islands and later to the Americas with the first confirmed cases of ZIKV reported in February 2015 in Brazil [2, 3]. Subsequently, ZIKV explosively spread across Latin American countries including the Caribbean [2]. In Curaçao and Aruba, ZIKV was detected from January 2016 to June 2017 [4–6]. Both islands also experienced dengue virus (DENV) circulation prior to and during the ZIKV outbreaks [7]. Little is known about the presence of other flaviviruses in Aruba and Curaçao. Prior reports of serological and molecular evidence in humans, bats, birds, and horses do indicate the presence of Saint Louis encephalitis virus (SLEV) and West Nile virus (WNV) in the Caribbean [8].

Although the number of (molecularly) confirmed cases during the ZIKV outbreaks in Aruba and Curaçao is known [4–6, 9], the actual magnitude of the outbreaks remains unclear since the majority of ZIKV cases are asymptomatic [10] and therefore likely not diagnosed. Additionally, symptomatic cases might have been missed as symptoms are generally mild and nonspecific [10]. After onset of clinical symptoms, there is a short window of time (~7 days in serum) in which ZIKV can be detected using molecular detection methods [11], likely further contributing to underreporting of ZIKV cases. Therefore, studying ZIKV seroprevalence may broaden our understanding of the extent of exposure during these outbreaks.

However, flaviviruses are known for inducing cross-reactive antibodies, which complicates serological analysis and differential diagnosis. Cross-reactive antibodies are preferentially boosted upon subsequent heterologous arbovirus infections explained by the phenomenon original antigenic sin (OAS) [12], and therefore especially complicate seroepidemiological studies in arbovirus (semi-)endemic regions. Antibody cross-reactivity has been attributed mostly to binding antibodies targeting the immunodominant envelope (E) protein [13]. The degree of cross-reactivity depends on the method used for testing, with highest specificity (and least cross-reactivity) reported for the use of virus neutralization tests (VNTs). Additionally, timing is important as it is thought that in the early phase after ZIKV infection, cross-neutralizing antibodies are transiently induced, whereas the late convalescent phase is characterized by less cross-reactivity together with the development of ZIKV-specific neutralizing antibodies, even in case of prior exposure to closely related DENV [14, 15]. These ZIKV-specific neutralizing antibodies likely target the more complex quaternary E protein epitopes spanning multiple domains across adjacent E protein dimers [13, 16].

Although VNTs are the gold standard for flavivirus serology, these assays are difficult to deploy; they require highly trained personnel and are time consuming and costly, thereby considered unfeasible in outbreak settings or for large datasets. As an alternative, detection of binding antibodies targeting the nonstructural protein 1 (NS1) can be used, since these antigens seem to be highly immunogenic as well as more specific to the infecting virus compared to other protein targets such as the E protein [17]. This increased specificity potentially allows for the use of binding antibody assays to study exposure to multiple flaviviruses.

This study aimed to determine the ZIKV seroprevalence in the early phase of, during, and after both ZIKV outbreaks on Curaçao and Aruba and thereby estimate the extent of exposure, using multiplex serology in combination with VNT confirmation assays. Therefore, we studied 2 longitudinal cross-sectional cohorts of sera obtained from healthy voluntary blood donors in Curaçao and Aruba during the ZIKV outbreaks (2016–2017). Furthermore, this study aims to complement our knowledge about the complex arbovirus antibody background patterns in highly endemic areas following arbovirus outbreaks.

## MATERIALS AND METHODS

### Study Design

This is a longitudinal cross-sectional study among the adult general population of Aruba and Curaçao. Blood donor sera were randomly selected from the Red Cross Blood Bank Foundation Curaçao and Horacio Oduber Blood Bank Aruba voluntary nonremunerated blood program collected between January 2016 and July 2017 on Curaçao (n = 395) and Aruba (n = 320) (~15 samples per month of each island). This time period included the ZIKV outbreaks on both islands. The program intake process only allows healthy individuals to regularly donate. Blood donor demographics were representative of all social classes dispersed over these Caribbean islands. The sample size selection was based on a power calculation (95% confidence level with a desired probability of .05) using the total population size in 2017 (Curaçao: 160 175; Aruba: 105 361) and estimated seroprevalence of 2%–5%, taking into account the number of (symptomatic) molecularly detected ZIKV cases documented by the Pan American Health Organization (PAHO) and diagnostic reports [4–6] (~600–800 cases in total), the percentage asymptomatic (~80%) versus symptomatic (~20%), and an expected increase of seroprevalence over time following the ZIKV outbreaks, as well as likely missed (mild) symptomatic cases. For Curaçao, additional metadata (age and sex) were available, as well as (additional) longitudinal donor samples (n = 72 donors, n = 157 longitudinal samples [2 or 3 timepoints per donor]). Longitudinal donor samples were used to study antibody kinetics and assess ZIKV seroconversion and/or antibody increase over time. Data on ZIKV polymerase chain reaction (PCR)–confirmed cases per month detected by the Public Health Department of Aruba and the Ministry of Health of Curaçao were used for comparison with the serological results presented in this study.

### Protein Microarray

Arbovirus immunoglobulin G (IgG) and immunoglobulin M (IgM) binding antibodies were detected using a protein microarray (PMA) as previously described with a few modifications [18–20]. For flaviviruses, NS1 antigens were printed on nitrocellulose glass slides: DENV-1–4 (DENV-1: Sino Biological, DENV-2–4: Immune Technology), ZIKV (Immune Technology), WNV (Sino Biological), tick-borne encephalitis virus (TBEV) (Immune Technology), SLEV (Immune Technology), yellow fever virus (YFV) (Immune Technology), Usutu virus (USUV) (The Native Antigen Company), and Japanese encephalitis virus (JEV) (Immune Technology). Slides were incubated with Blocker Blotto in tris-buffered saline (Thermo Fisher Scientific) to prevent nonspecific antibody binding. IgG antibodies in serial 4-fold diluted sera (1:20 to 1:1280) were detected by incubation with secondary antibody AlexaFluor 647–conjugated goat-antihuman IgG-Fcγ (Jackson Immunoresearch). IgM was detected in 1:20 diluted sera in GullSORB (Meridian Bioscience) for inhibition of IgG antibodies, followed by incubation with AlexaFluor 647–conjugated goat-antihuman IgM-Fc_5µ_ (Jackson Immunoresearch). Phosphate-buffered saline 0.05% Tween 20 washing buffer (Sigma-Aldrich) was used for washing between incubation steps. The Tecan PowerScanner was used for scanning the slides and ImaGene 9.0 software (Biodiscovery) was used to analyze antigen-antibody binding by using fluorescence intensity of individual antigen spots. The median fluorescence of 2 identical protein spots was used to calculate 50% maximal effective concentration IgG titers. Serum samples with IgG titers ≥20 and IgM median fluorescence intensity signals ≥20 000 were considered to be test reactive.

### Virus Neutralization Test

To define a cut-off for the PMA signals, a selection of serum samples (n = 82) with likely ZIKV exposure (ZIKV/DENV NS1 ratio >1, ZIKV IgM positive and/or clear ZIKV IgG kinetics [>2-fold increase, given the expected presence of cross-reactive background antibodies and taking into account OAS [21]], or maximum titers over time), as well as some nonreactive samples, were tested by confirmatory VNTs. ZIKV- and DENV-specific neutralizing antibodies were measured using in-house VNTs [20]. One hundred 50% tissue culture infectious dose (TCID50) of DENV-2 [22] or the ZIKV Suriname strain (2016, GenBank reference KU937936) was incubated with serial diluted serum (2-fold, triplicate testing) on a confluent monolayer of Vero cells in 96-well plates (1 hour, 37°C, 5% carbon dioxide). Washing of cells was done 3 times and cells were subsequently incubated with Dulbecco's modified Eagle medium supplemented with 10% fetal bovine serum for 5 days (ZIKV) or 7 days (DENV-2) for the detection of cytopathic effect. The highest final serum dilution that completely prevented the cytopathic effect was used to calculate the geometric mean. We used a positive cut-off of >1:32 based on validation datasets that included a cross-reactivity flavivirus sera panel of serum samples from persons with known vaccination or infection exposure history (YFV, TBEV, WNV and JEV) [20]. Confirmed ZIKV exposure was defined by a positive ZIKV VNT titer that exceeded the DENV VNT titer by >4-fold.

### Statistical Analysis and Seroprevalence Estimation

Trend assessment of IgG titers across age groups was done by calculating *P* values for trend lines using the Jonckheere-Terpstra test in R software version 4.2.2. To estimate ZIKV IgG seroprevalence before and after the ZIKV outbreak, VNT titers of a selection of sera (suspected ZIKV and negative samples; n = 72 for Curaçao; n = 10 for Aruba) were used to determine the PMA cut-off for the detection of ZIKV-specific IgG binding antibodies. To determine the PMA cut-off for ZIKV NS1 that best describes ZIKV-specific antibodies rather than cross-reactive antibodies, a receiver operating characteristic (ROC) curve analysis was done taking into account the ZIKV-DENV VNT ratio groups as well as the ZIKV VNT values. Since in endemic areas, individuals likely have an antibody background from prior exposure(s), consisting of both specific and cross-reactive antibodies, VNT assays may not be able to fully discriminate ZIKV from DENV when priorly exposed to DENV. Therefore, we chose a ZIKV NS1 cut-off at 100% sensitivity with the best possible specificity, to include all potential ZIKV-exposed individuals in further analyses.

## RESULTS

### Antibody Levels and Age

**Figure 1.**
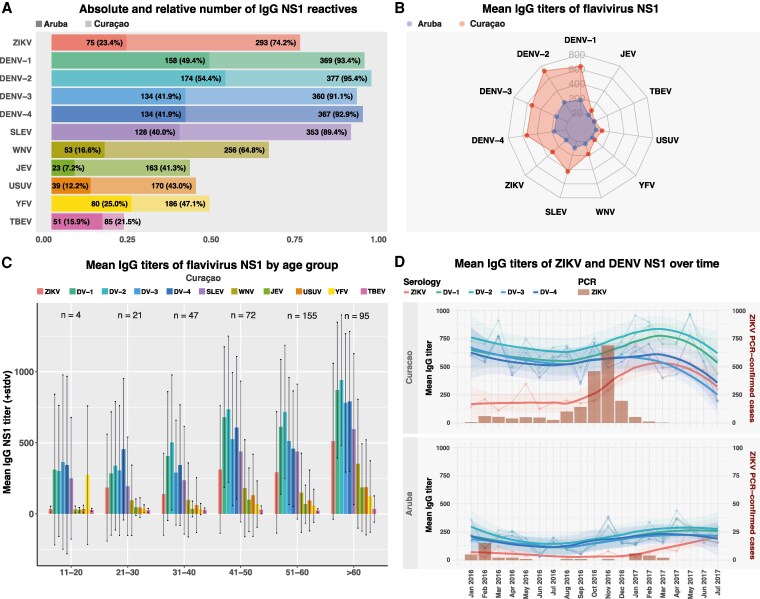
Immunoglobulin G (IgG) nonstructural protein 1 (NS1) protein microarray reactivity for Curaçao and Aruba. *A*, Absolute and relative number of sera reactive for flavivirus IgG NS1; colors represent the different antigens for blood donor sera samples. Dark-shaded colors depict the counts for Aruba (n = 320) and light-shaded colors depict the counts for Curaçao (n = 395). *B*, Mean flavivirus IgG titers in Aruba and Curaçao. *C*, Mean IgG flavivirus NS1 titers and standard deviation across age groups for Curaçao (n = 394). One participant was excluded due to missing data regarding age. *D*, Mean Zika virus and dengue virus IgG NS1 titers (linear and smoothed line) and polymerase chain reaction–confirmed case counts (barplots) from January 2016 until July 2017 for Curaçao (upper panel) and Aruba (lower panel). Abbreviations: DENV, dengue virus; DV, dengue virus; IgG, immunoglobulin G; JEV, Japanese encephalitis virus; NS1, nonstructural protein 1; PCR, polymerase chain reaction; SLEV, Saint Louis encephalitis virus; stdv, standard deviation; TBEV, tick-borne encephalitis virus; USUV, Usutu virus; WNV, West Nile virus; YFV, yellow fever virus; ZIKV, Zika virus.

We drew a random sample of 395 sera from Curaçao and 320 sera from Aruba (~15 samples per month of each island) from donations collected between January 2016 and July 2017 and stored by the local blood banks. The majority of blood donors from both islands had IgG antibodies to multiple flaviviruses detected by PMA, of which IgG antibody titers toward DENV-1–4 were most frequently detected (Figure 1*A*). The number of IgG-reactive donors and mean IgG titers for Curaçao were considerably higher than in Aruba against all tested viruses (Figure 1*A* and 1*B*). The mean IgG titers for DENV-1–4 were highest on both islands, with DENV-2 consistently showing the highest titers followed by DENV-1, DENV-4, and DENV-3, subsequently followed by SLEV and ZIKV (Figure 1*B*). Limited IgG reactivity was found toward viruses that are not endemic in Latin America (JEV, USUV, TBEV) [23] (Figure 1*A* and 1*B*), therefore likely reflecting antibody cross-reactivity from other flavivirus exposures. Mean IgG titers generally increased by age for flaviviruses in Curaçao (Figure 1*C* and Supplementary Figure 1) (trend line *P* < .05).

### Antibody Kinetics

ZIKV-NS1 IgG antibody levels from sera collected over time show distinct fluctuations correlating with the known epidemiological curves (epi-curves) of the ZIKV outbreaks on both islands reported by PAHO [5, 6], and trends of ZIKV PCR-confirmed cases per month detected by the Public Health Department of Aruba and the Ministry of Health of Curaçao (Figure 1*D*). The levels of antibodies to other flaviviruses, especially DENV-1–4 and SLEV, showed a clear increase at the same time, likely explained by boosting of cross-reactive antibodies (Figure 1*D* and Supplementary Figure 2). The ZIKV IgG antibody increase was higher in Curaçao compared to Aruba, in line with PAHO observations as well as the PCR results (Figure 1*D*).

**Figure 2.**
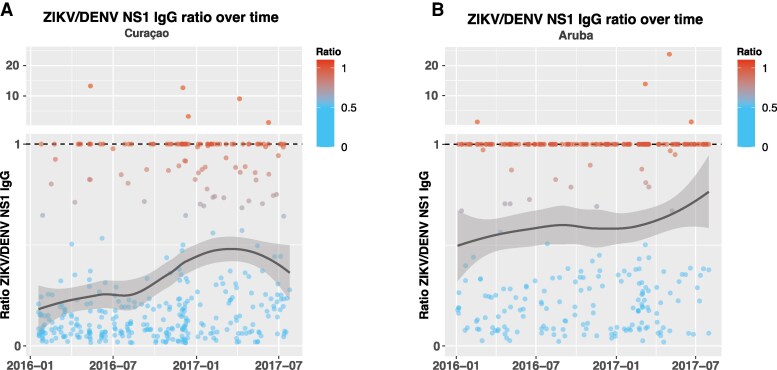
Immunoglobulin G (IgG) titer ratio of Zika virus (ZIKV)/dengue virus (DENV; subtypes 1–4) nonstructural protein 1 (NS1) over time for Curaçao (*A*) and Aruba (*B*). A regression line with confidence intervals was added to the plot to illustrate the ratio value trend over time.

**Figure 3.**
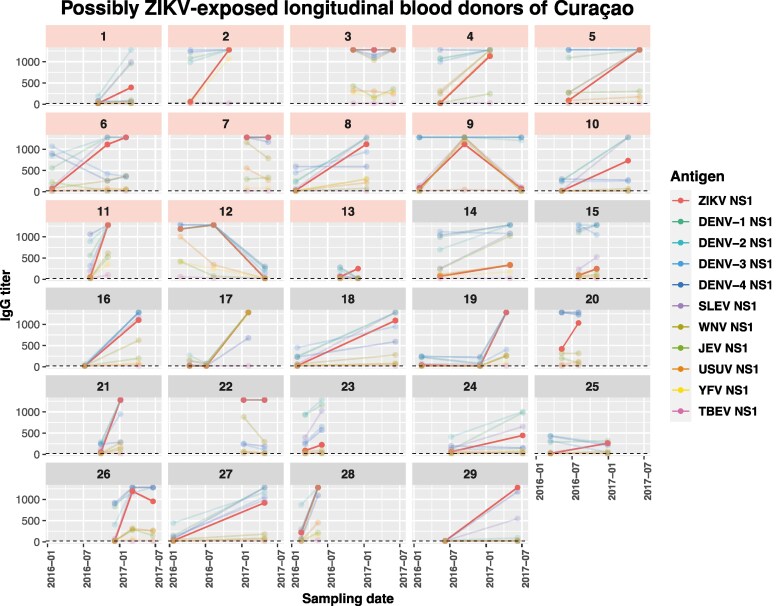
Flavivirus nonstructural protein 1 immunoglobulin G over time of longitudinal blood donors (2 or 3 timepoints) that possibly reflect Zika virus (ZIKV) exposure (N = 29). Donors 1–13 are ZIKV confirmed based on the virus neutralization test (pink panels). Abbreviations: DENV, dengue virus; IgG, immunoglobulin G; JEV, Japanese encephalitis virus; NS1, nonstructural protein 1; SLEV, Saint Louis encephalitis virus; TBEV, tick-borne encephalitis virus; USUV, Usutu virus; WNV, West Nile virus; YFV, yellow fever virus; ZIKV, Zika virus.

Only a few sera (Aruba: 4/320 [1.3%]; Curaçao: 5/395 [1.3%]) had higher IgG titers for ZIKV compared to DENV-1–4 (Figure 2*A* and 2*B*). However, the ZIKV/DENV NS1 ratio increased over time (Figure 2*A* and 2*B*), indicating a rise in ZIKV IgG antibodies and decreasing difference between ZIKV and DENV titers. Of 72 donors with longitudinal samples (2 or 3 timepoints) (Supplementary Figure 3), 29 donors showed a medium to high ZIKV IgG NS1 titer (>100) in combination with a clear increase (>2-fold) or maximum ZIKV-NS1 IgG titer over time (Figure 3), suggesting (recent) ZIKV exposure. Titers against other flaviviruses, generally SLEV (25/29 [86.2%]), DENV-1 (23/29 [79.3%]), and WNV (22/29 [75.9%]), often increased as well (Figure 3 and Supplementary Table 1). Although blood donors often had high DENV titers (>100) at the first timepoint (27/29 [93.1%]) and about one-third showed a 4-fold increase in titer over time (DENV-1: 11/29 [37.9%]; DENV-2: 8/29 [27.6%]; DENV-3: 7/29 [24.1%]; DENV-4: 6/29 [20.7%]), a considerable proportion also had a >4-fold increase in antibody levels for other flavivirus antigens including ZIKV (22/29 [75.9%]), SLEV (21/29 [72.4%]), and WNV (14/29 [48.3%]) (Figure 3 and Supplementary Table 1). Only a few donor samples (Aruba: 2/320 [0.6%]; Curaçao: 5/395 [1.3%]) had ZIKV-NS1 IgM antibodies (Supplementary Figure 4). These donors with ZIKV IgM–positive sera were generally detected during the peaks in PCR-detected ZIKV cases.

### Confirmation of ZIKV Exposure and Estimated ZIKV Seroprevalence

**Figure 4.**
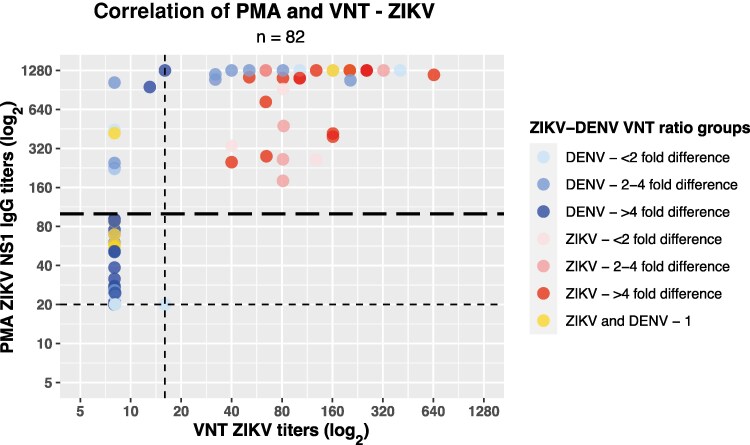
Discrimination of Zika virus (ZIKV) and dengue virus (DENV) by virus neutralization test (VNT). Correlation of ZIKV nonstructural protein 1 (NS1) immunoglobulin G (IgG) titers of protein microarray (PMA) with ZIKV VNT titers for Curaçao (n = 72) and Aruba (n = 10). Dotted lines represent the cut-off values of the PMA and VNT. The long-dashed line represents the PMA cut-off (100) that best distinguishes ZIKV and DENV signals. ZIKV-DENV VNT ratio groups are represented in colors: <2, 2–4, or >4 fold difference with ZIKV for positive DENV titers (blue shades), with DENV for positive ZIKV titers (red shades), or no difference in VNT titer between ZIKV and DENV (yellow).

**Figure 5.**
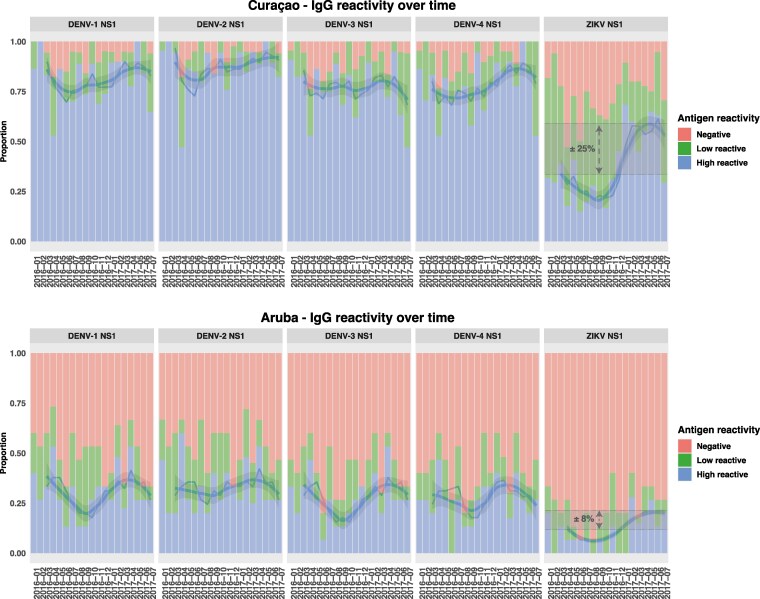
Estimated Zika virus (ZIKV) seroprevalence. Percentage barplots of immunoglobulin G (IgG) reactivity over time for Curaçao and Aruba. The barplot colors represent antigen IgG titers (negative, <20; low reactive, 20–100; high reactive, >100). The plotted line shows the rolling average of the high reactives (k = 3) with the corresponding confidence interval (gray). Dotted lines and arrows show the estimated ZIKV seroprevalence percentages. Abbreviations: DENV, dengue virus; IgG, immunoglobulin G; NS1, nonstructural protein 1; ZIKV, Zika virus.

VNT assays were performed on the samples possibly reflecting ZIKV exposure (ZIKV/DENV NS1 ratio >1, ZIKV IgM positive, and/or clear ZIKV IgG kinetics or maximum titers over time; Aruba: n = 6 donors; Curaçao: n = 36 donors), as well as some nonreactive samples, showing a distinction of ZIKV versus DENV in VNT assays (>4-fold difference) (Figure 4). This resulted in 15 confirmed ZIKV cases out of 36 selected donors (41.7%) tested in the VNT in Curaçao, which was 13 of 29 (44.8%) selected longitudinal donors (Figure 3, pink panels). For Aruba, VNT assays confirmed 1 of 6 (16.7%) suspected ZIKV donors in total. The ZIKV-DENV distinction was most optimal when using a ZIKV NS1 PMA cut-off of 100 based on ROC analysis (Figure 4 and Supplementary Figure 5), including all donors with higher ZIKV VNT titers than DENV, even though this possibly includes some false positives (Figure 4 and Supplementary Figure 5). Using this cut-off, we categorized PMA signals into 3 groups (negative, <20; low reactive, 20–100; high reactive, >100) to estimate ZIKV seroprevalence before and after the ZIKV outbreak. Despite a likely overrepresentation due to antibody cross-reactivity, seroprevalence increase of Curaçao after the study period was estimated to be around 25% (24.7% [95% confidence interval {CI}, 21.0%–28.3%]), taking into account the antibody background levels at the start of the study, whereas for Aruba this was around 8% (8.1% [95% CI, 7.2%–9.0%]) (Figure 5).

## DISCUSSION

The ZIKV outbreak in Latin America and the Caribbean has led to >800 000 suspected and confirmed cases by the end of 2017 [24]. Studying ZIKV seroprevalence and understanding human ZIKV infection in a highly endemic flavivirus background in an immunological context may complement our knowledge about (boosting of) antibody cross-reactivity and overall antibody (background) patterns following flavivirus exposure. This may give insights into the (immunological) behavior of possible future flavivirus outbreaks in similar endemic areas as well as associated diagnostic challenges and (sero)surveillance. This knowledge is also important to allow interpretation of vaccination responses, including potential unintended effects as was observed during the population-level application of the first DENV vaccine [25]. Here, in a group of children who had their first exposure by the DENV vaccine, enhanced severity of disease was observed when they encountered their first natural infection after vaccination, due to a phenomenon called antibody-dependent enhancement [25].

In this study, we show the use of biobanked sera from blood banks for studying infectious disease outbreaks. Despite a high DENV IgG antibody background in blood donors, we were able to estimate the ZIKV seroprevalence increase, which was higher in Curaçao (~25%) (~8%) at the end of the study period in July of 2017. This is in line with PAHO documentation based on clinical notifications, describing a higher frequency of total cases in Curaçao [5, 6, 24]. Furthermore, ZIKV IgG NS1 antibody kinetics and seroprevalence patterns correlate with the known epi-curves of the ZIKV outbreaks in Curaçao and Aruba [4–6, 9]. The different seroprevalence as well as the different total number and timing of detected cases on the islands do not seem to be explained by tourism patterns; both islands had a similar travel influx from South America (~20%) in 2016 and 2017, especially from Venezuela, Colombia, and Brazil [26–28]. The islands did have differences in the number of tourists from North America and Europe, with around 60%–70% of tourists from North America in 2016 and 2017 for Aruba, whereas this was only around 20% for Curaçao with most tourists from Europe (~50%), mainly the Netherlands [26–28]. Differences in mosquito species abundance, vector control, and/or slightly different climate conditions could possibly have influenced the epi-curve patterns on the islands; however, not much data are available for these possible risk factors [29–31], also highlighting the need for further research.

The already high ZIKV and DENV seroprevalence in Curaçao in January 2016, when the first ZIKV cases were reported by PAHO, might be explained by an earlier undetected start of the ZIKV outbreak in 2015. Another explanation is the past DENV circulation in Curaçao that could have led to a high DENV seroprevalence at the beginning of the study period [32], as well as a high ZIKV seroprevalence from antibody cross-reactivity [33]. Boosting of the baseline cross-reactive ZIKV antibody levels due to ZIKV exposure can therefore also be the reason for the higher observed seroprevalence at the end of the study in Curaçao compared to Aruba, as well as the higher mean (peak) IgG titers against ZIKV NS1 over time for Curaçao. However, evidence of potential differences in the extent of DENV circulation between Curaçao and Aruba in the years prior to the ZIKV outbreak is limited, and possibly biased by lack of surveillance and/or documentation [7].

The observed high DENV IgG antibody background in blood donors—a high number of PMA IgG DENV NS1 reactive blood donor sera as well as high mean IgG titers against DENV-1–4—was mostly directed toward DENV-2, followed by DENV-1, DENV-4, and DENV-3. All 4 DENV subtypes are known to have been circulating in Aruba and Curaçao, of which, although limited information is available from Aruba and Curaçao [34–36], DENV cases in the prior 5 years (2011–2016) seem to have been largely caused by the DENV-1, -2, and -4 subtypes. The overall flavivirus IgG background was more prominent in Curaçao compared to Aruba, suggesting differences in past flavivirus circulation. Next to DENV and ZIKV, IgG NS1 titers for SLEV and WNV were also frequently detected. This may be explained by cross-reactive antibodies induced upon DENV and/or ZIKV infection [14, 37], but might also in part be reflected by true (past) exposures. Considering the knowledge gap of SLEV and WNV circulation on Aruba and Curaçao [8], further research is necessary to acquire a better understanding of the prevalence of these viruses in the region. Similar antibody-specific and cross-reactive (boosting) patterns were seen across increasing donor age and in longitudinal sera of ZIKV suspected cases, likely explained by OAS and the antigenic distance between the studied flaviviruses, and thereby priming of antibodies upon (multiple sequential) flavivirus exposures [7]. The low number of ZIKV IgM–reactive sera for Curaçao (n = 5) and Aruba (n = 2) in this study may be explained by the timing of testing and (rapid) waning of IgM antibodies [38, 39]. Additionally, literature suggests that in a flavivirus-endemic area—in the case of individuals who have been exposed to (multiple) flaviviruses in the past—induction of IgM might be less potent or even absent [40].

This study shows the utility and importance of an existing voluntary blood donor repository in endemic areas, often developing countries, to complement (retrospective) outbreak investigations as well as facilitating the potential (early) detection of outbreaks. This is especially relevant for those concerning virus infections with a relatively large proportion of asymptomatic cases, and particularly in the absence of other effective national surveillance systems. Existing blood donor systems also proved their utility in Brazil to monitor arbovirus circulation in a highly endemic area and its potential use in outbreak situations [41, 42], taking into account RNA detection and/or IgM (likely) reflecting recent infection. Remaining challenges are the short window for RNA detection, sample storage conditions, and obtaining appropriate follow-up samples for confirmation of infection, as well as the serological challenges of studying closely related viruses within a high arbovirus background, especially when using single-antigen serological tests. Currently, the introduction and use of voluntary blood donor programs is often underdeveloped in low- and middle-income (flavivirus-endemic) countries compared to high-income countries [43, 44]. This is likely explained by absence of effective national blood programs, resulting in insufficient number of (volunteer) donors and insufficient availability of safe, high-quality labile blood (products).

In summary, we show that likely many more individuals were infected by ZIKV during the outbreaks in Curaçao and Aruba compared to the number of PAHO-reported (molecular) detections [5, 6]. Furthermore, this study gives insights into the complex antibody-specific and cross-reactive (boosting) patterns in flavivirus-exposed individuals, and demonstrates the value of using multiantigen serology in distinguishing specific antibodies from a high-antibody background. Estimating population immunity levels is crucial for assessing the risk and possible outcomes of future outbreaks involving closely related viruses (eg, ZIKV or DENV), particularly in evaluating the balance between protective immunity and the potential for antibody-dependent enhancement of infection [45–48], and in the context of evaluating potential vaccination programs.

## Supplementary Material

jiaf281_Supplementary_Data

## Supplementary Data

Supplementary materials are available at *The Journal of Infectious Diseases* online (http://jid.oxfordjournals.org/). Supplementary materials consist of data provided by the author that are published to benefit the reader. The posted materials are not copyedited. The contents of all supplementary data are the sole responsibility of the authors. Questions or messages regarding errors should be addressed to the author.

## Notes

***Author contributions.*** Conceptualization: L. M. R. K., R. S. S., I. G., A. D., and M. P. G. K. Data curation and formal analysis: L. M. R. K. Funding acquisition: I. G., A. D., and M. P. G. K. Investigation: L. M. R. K. and F. C. Methodology: L. M. R. K., R. S. S., F. C., J. H. G. v. B., I. G., A. D., and M. P. G. K. Project administration: I. G., A. D., and M. P. G. K. Resources: J. H. G. v. B., Y. H., N. L., E. G. M., I. G., A. D., and M. P. G. K. Software and visualization: L. M. R. K. Supervision: R. S. S. and M. P. G. K. Validation: L. M. R. K., F. C., R. S. S., and M. P. G. K. Writing–original draft: L. M. R. K. Writing–review and editing: All authors.

***Acknowledgments.*** We would like to thank the Dutch Caribbean preparedness for mosquito-borne infectious diseases (DUCAMID) applicants for study set-up and funding acquisition, all voluntary blood donors who participated in this study, and everyone involved in the sampling and data collection of the included blood donors at the blood bank. Furthermore, we would like to thank everyone involved in the transport of blood donor samples to Erasmus Medical Center for serological analysis, and everyone from the Public Health Department of Aruba and Ministry of Health of Curaçao involved in the molecular analysis of ZIKV cases during the 2016–2017 ZIKV outbreaks.

***Financial support.*** This study was part of the DUCAMID project (ALWCA.2016.039), funded by the Dutch Research Council (NWO).
