# Supplementary material for: Estimating Zika Virus Seroprevalence in a Dengue Virus–Endemic Population: The Use of Blood Donors and Multiplex Serology to Monitor Arbovirus Outbreaks in the Dutch Caribbean
Source: J Infect Dis. 2025 May 27;232(3):e507–17. doi: 10.1093/infdis/jiaf281 (PMC12455303; doi:10.1093/infdis/jiaf281)
Supplement: jiaf281_Supplementary_Data [file jiaf281_supplementary_data.docx]

**Supplement**


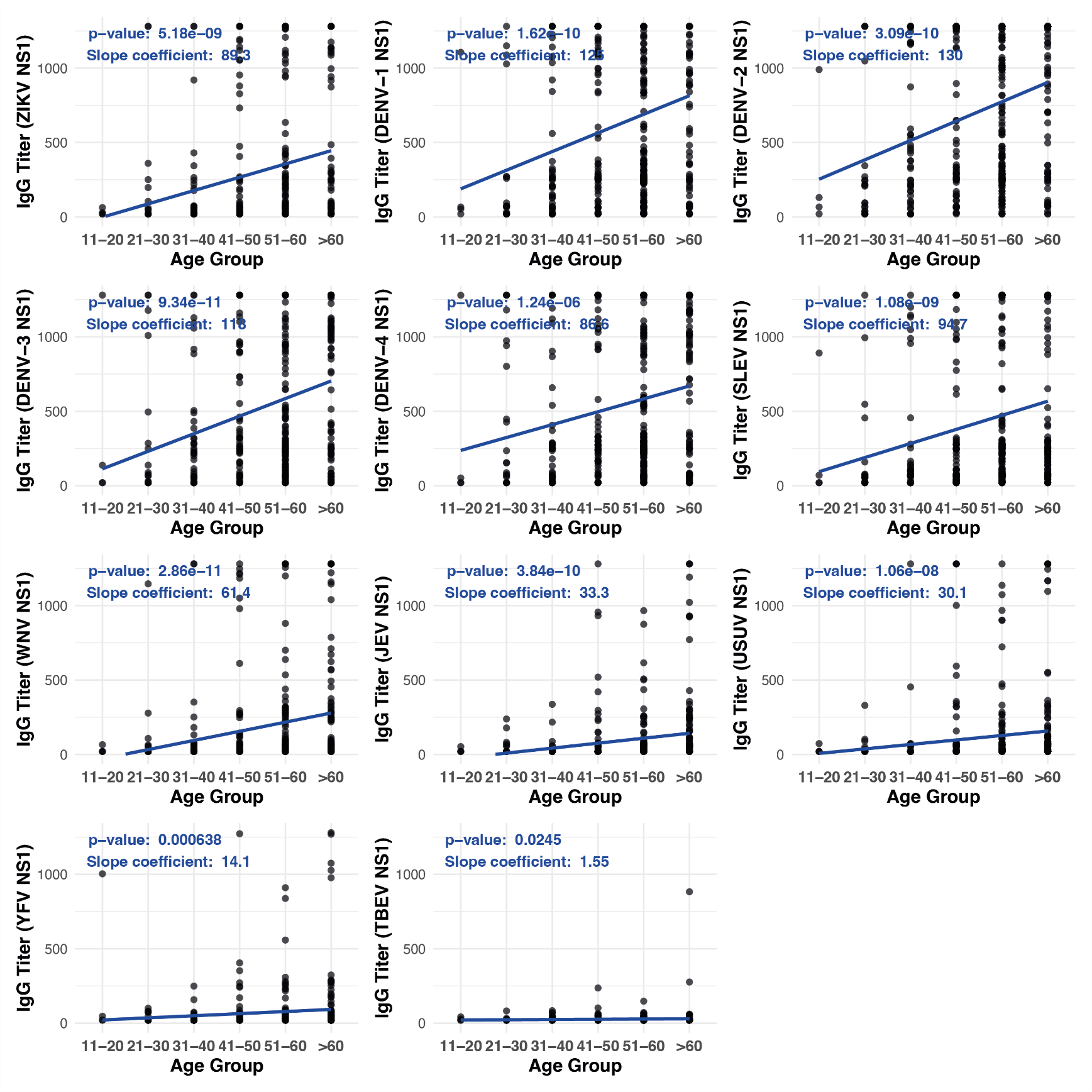


**Figure S1 – Trends in flavivirus NS1 IgG titers across age groups in Curacao**Trend lines are shown for IgG antibodies towards each antigen across the different age groups. P-values and slope coefficients are shown in blue.


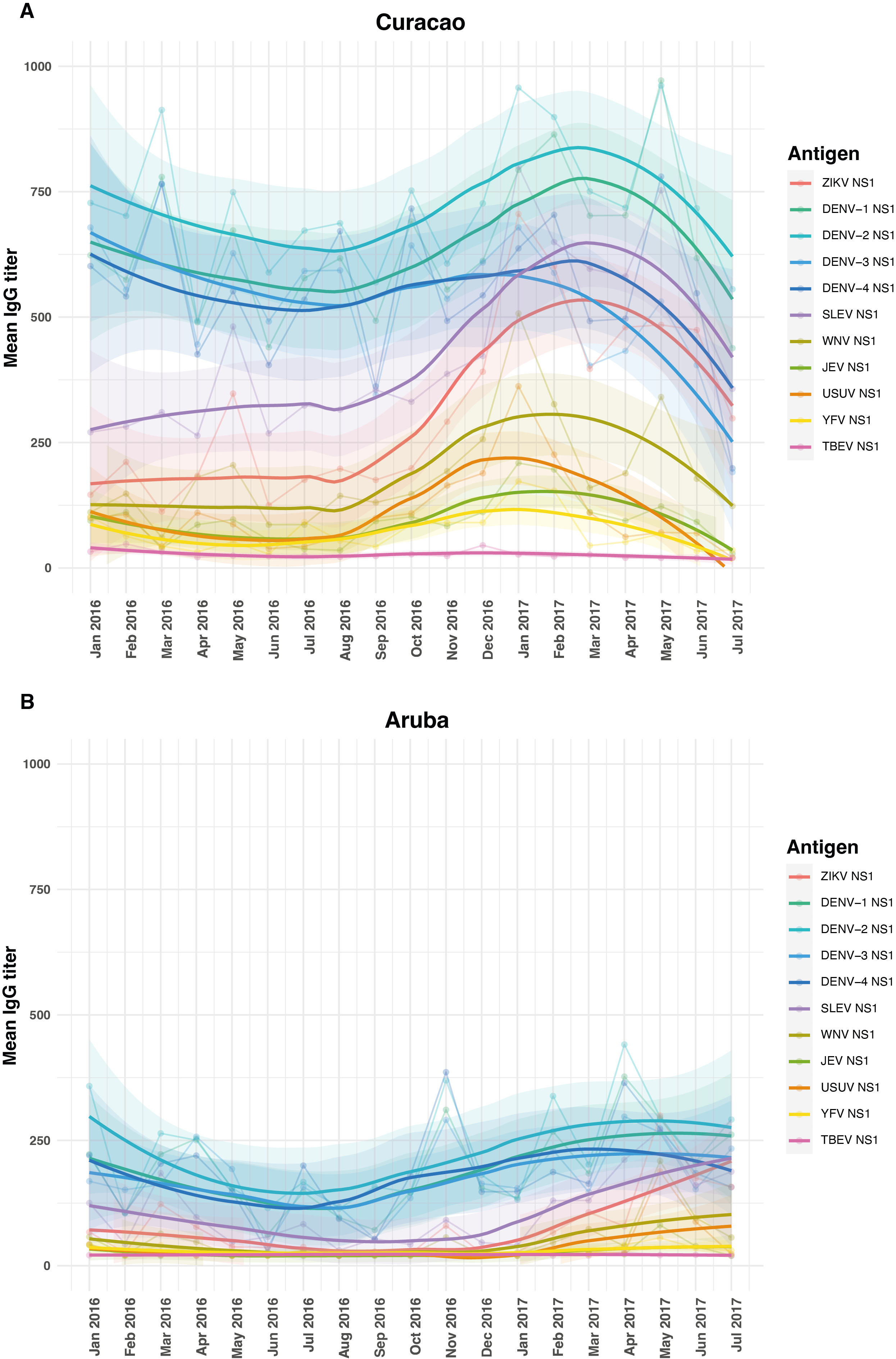


**Figure S2 – Mean flavivirus NS1 IgG titers over time**

**Figure S3 – Flavivirus NS1 titers of longitudinal donor samples**Total N = 72 longitudinal donors with two or three timepoints.

**Figure S4 – ZIKV NS1 IgM fluorescence signal over time**A) Curacao, B) Aruba


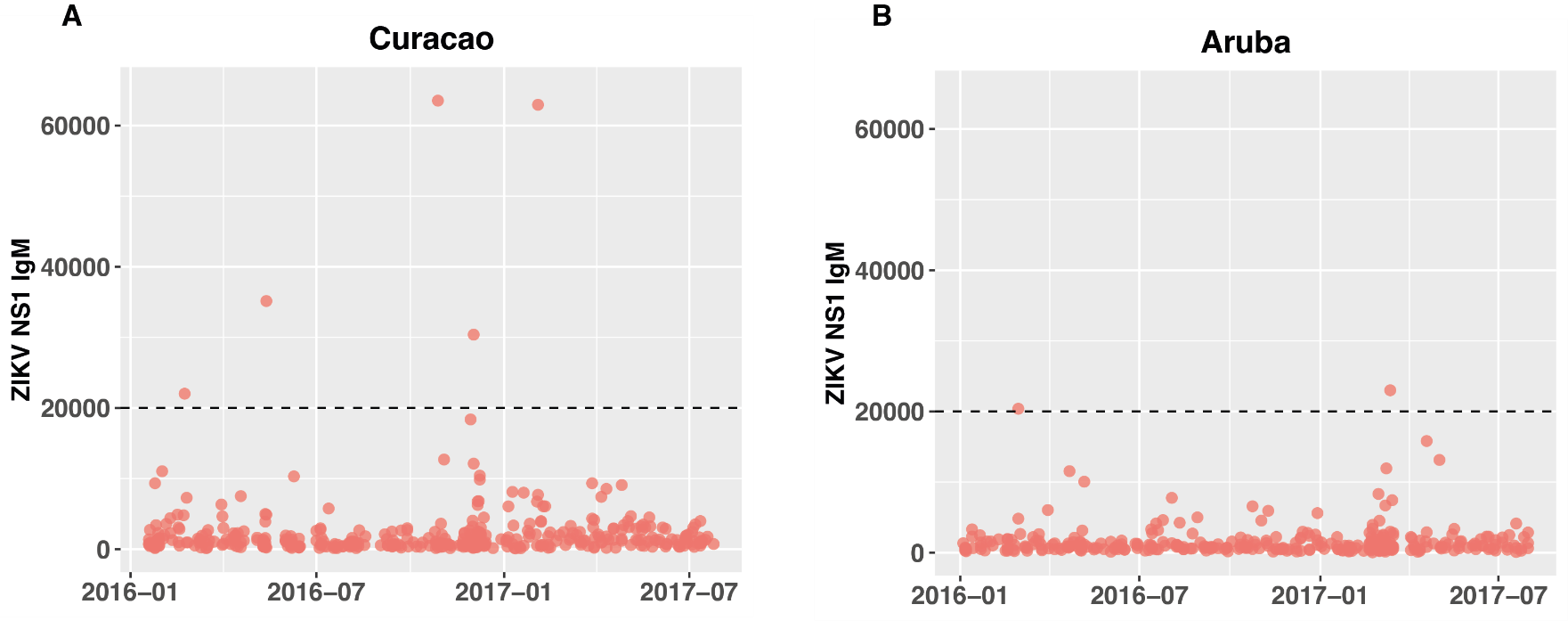


**Figure S5 – ROC curve for ZIKV NS1 cut-off**A ROC curve was calculated to determine the PMA cut-off for ZIKV NS1 that best describes ZIKV-specific antibodies. ZIKV-DENV VNT ratio groups of ZIKV - <2 fold difference, ZIKV - 2-4 fold difference and ZIKV - >4 fold difference in combination with ZIKV VNT signals equal to or above titer 16 (VNT cut-off), were considered as a ZIKV case, whereas the ZIKV-DENV VNT ratio groups DENV - <2 fold difference, DENV - 2-4 fold difference, DENV - >4 fold difference, ZIKV and DENV – 1 and/or ZIKV VNT signals <16 were categorized as non-ZIKV. Specificity and sensitivity values are shown for the optimal ZIKV NS1 PMA cut-off of 100.


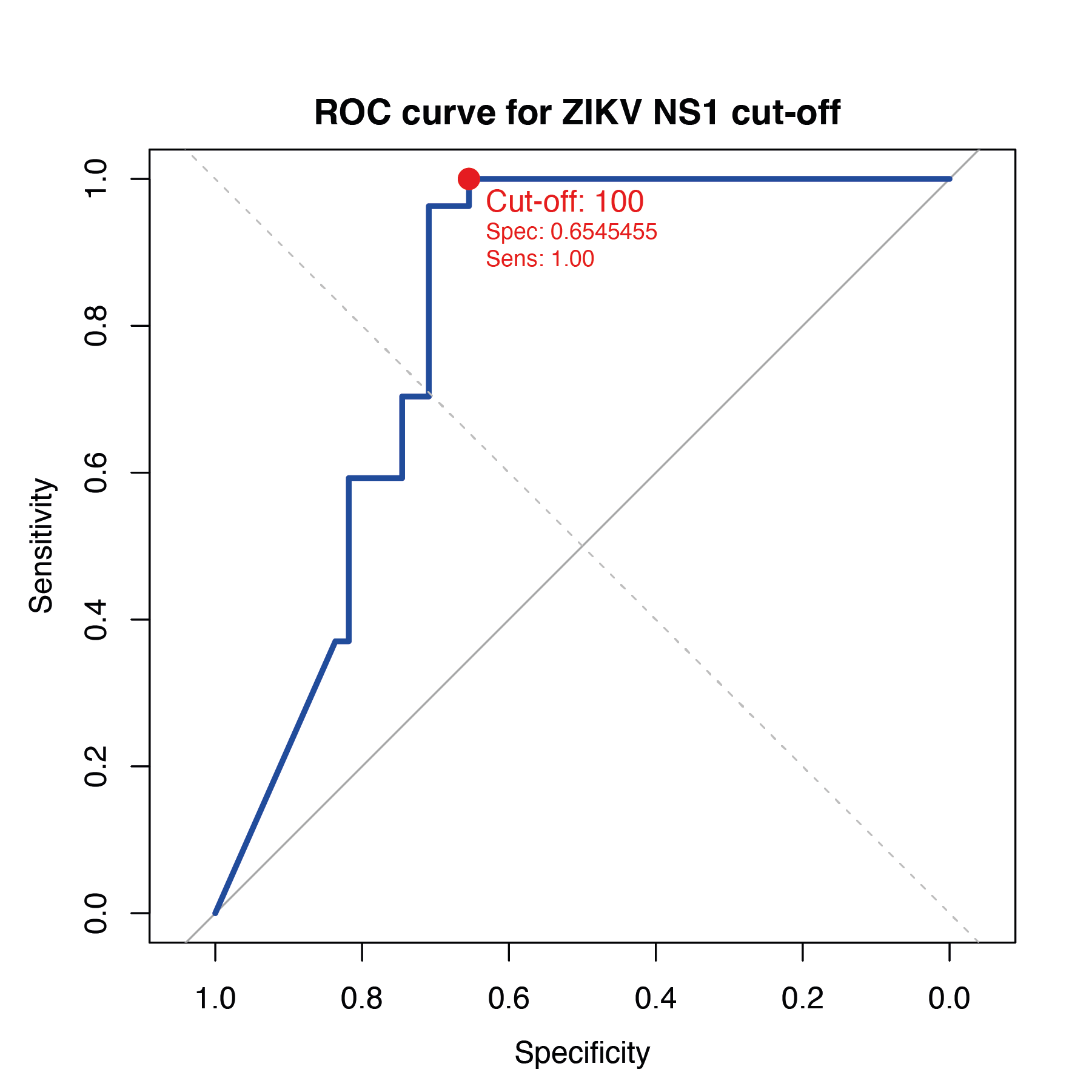


**Table S1 – Frequency of flavivirus NS1 IgG titer increase for ZIKV suspected longitudinal donors**Percentages show the relative IgG (fourfold-)increase count calculated upon the total number of ZIKV suspected cases (longitudinal donors, n = 29).

| **Antigen** | **Increase count** | **Four-fold increase count** | **Total ZIKV suspected** |
| --- | --- | --- | --- |
| **DENV-1 NS1** | 23 (79.3%) | 11 (37.9%) | 29 |
| **DENV-2 NS1** | 20 (69.0%) | 8 (27.6%) | 29 |
| **DENV-3 NS1** | 15 (51.7%) | 7 (24.1%) | 29 |
| **DENV-4 NS1** | 15 (51.7%) | 6 (20.7%) | 29 |
| **SLEV NS1** | 25 (86.2%) | 21 (72.4%) | 29 |
| **WNV NS1** | 22 (75.9%) | 14 (48.3%) | 29 |
| **YFV NS1** | 20 (69.0%) | 6 (20.7%) | 29 |
| **USUV NS1** | 20 (69.0%) | 10 (34.5%) | 29 |
| **TBEV NS1** | 7 (24.1%) | 1 (3.4%) | 29 |
| **JEV NS1** | 21 (72.4%) | 12 (41.4%) | 29 |
| **ZIKV NS1** | 26 (89.7%) | 22 (75.9%) | 29 |
